# Trends in Ischemic Stroke Hospitalization and Outcomes in the United States Pre- and Peri-COVID-19 Pandemic: A National Inpatient Sample Study

**DOI:** 10.3390/jcm14041354

**Published:** 2025-02-18

**Authors:** Alibay Jafarli, Mario Di Napoli, Rachel S. Kasper, Jeffrey L. Saver, Louise D. McCullough, Setareh Salehi-Omran, Behnam Mansouri, Vasileios Arsenios Lioutas, Mohammed Ismail, Afshin A. Divani

**Affiliations:** 1 Department of Neurology, University of Texas, San Antonio, TX 78712, USA; jafarli@uthscsa.edu; 2 Neurological Service, Dell’annunziata Hospital, 67039 Sulmona, L’Aquila, Italy; mariodinapoli@katamail.com; 3 Department of Neurology, University of New Mexico, Albuquerque, NM 87106, USA; rkasper@salud.unm.edu (R.S.K.); mohismail@salud.unm.edu (M.I.); 4 Comprehensive Stroke Center and Department of Neurology, University of California, Los Angeles (UCLA), Los Angeles, CA 90095, USA; jsaver@mednet.ucla.edu; 5 Department of Neurology, McGovern Medical School, The University of Texas at Houston, Houston, TX 77082, USA; louise.d.mccullough@uth.tmc.edu; 6 Department of Neurology, University of Colorado School of Medicine, Aurora, CO 80045, USA; setareh.salehiomran@cuanschutz.edu; 7 Department of Neurology, Shahid Beheshti University of Medical Sciences, Tehran 1983963113, Iran; bsmansouri@gmail.com; 8 Department of Neurology, Beth Israel Deaconess Medical Center, Harvard Medical School, Boston, MA 02215, USA; vlioutas@bidmc.harvard.edu

**Keywords:** ischemic stroke, outcomes, hospitalization costs, trends, COVID-19, National Inpatient Sample, complications, mechanical thrombectomy, thrombolysis

## Abstract

**Background/Objectives:** The COVID-19 pandemic impacted healthcare systems globally, disrupting the management and treatment of acute ischemic stroke (AIS). Understanding how AIS admissions, treatments, and outcomes were affected is critical for improving stroke care in future crises. The objective of this work was to assess the COVID-19 pandemic’s impact on AIS admissions, treatment patterns, complications, and patient outcomes in the U.S. from 2016 to 2021, focusing on differences between pre-pandemic (2016–2019) and peri-pandemic (2020–2021) periods. **Methods:** This is a retrospective cohort study using the National Inpatient Sample (NIS) database, analyzing weighted discharge records of AIS patients over six years. Data encompass U.S. hospitals, including urban, rural, teaching, and non-teaching facilities. The study included AIS patients aged 18 and older (N = 3,154,154). The cohort’s mean age was 70.0 years, with an average hospital stay of 5.1 days and an adjusted mean cost of $16,765. Men comprised 50.5% of the cohort. We analyzed temporal trends in AIS hospitalizations from 2016 to 2021, comparing pre- and peri-COVID-19 periods. The primary outcome was the AIS admissions trend over time, with secondary outcomes including reperfusion therapy utilization, intubation rates, discharge disposition, and complications. Trends in risk factors and NIH Stroke Scale (NIHSS) severity were also evaluated. **Results:** AIS admissions rose from 507,920 in 2016 to 535,694 in 2021. Age and sex distribution shifted, with a growing proportion of male AIS cases (from 49.8% to 51.4%) and a decrease in mean age from 70.3 to 69.7 years. Although not statistically significant, White patients were the majority (68.0%), though their proportion declined as Black, Hispanic, and Asian/Pacific Islander cases increased. Reperfusion therapy, especially mechanical thrombectomy, rose from 2.2% to 5.6% over the study period. Intubation rates increased from 4.8% pre-COVID-19 to 5.5% peri-COVID, with higher rates among COVID-positive patients. NIHSS severity declined over time, with severe strokes (NIHSS ≥ 16) decreasing from 14.5% in 2017 to 12.6% in 2021. **Conclusions:** The COVID-19 pandemic brought significant shifts in AIS patterns, with younger, more diverse patients, increased reperfusion therapy use, and rising complication rates. These changes underscore the importance of resilient healthcare strategies and resource allocation to maintain stroke care amid future public health emergencies.

## 1. Introduction

Stroke remains a leading cause of death and disability globally and is the fifth leading cause of death in the United States (U.S.) [1,2,3]. Advances in acute ischemic stroke (AIS) care, including intravenous thrombolysis (IVT) and mechanical thrombectomy (MT), have improved outcomes, especially with prompt administration [4,5]. These interventions have reduced long-term disability, increased survival, and improved chances of favorable functional outcomes. Despite these advances, disparities in stroke rates by age, race, and region remain [6,7,8,9,10,11]. Age remains an independent risk factor for stroke, contributing to an increasing incidence and prevalence of stroke among the elderly population [9,10]. Regionally, the “stroke belt” in the southeastern U.S. continues to exhibit disproportionately higher stroke incidence and prevalence compared to other regions of the country, likely due to a combination of lifestyle, genetic, and healthcare access factors [6]. These disparities underscore the urgent need for tailored public health interventions aimed at high-risk populations, including preventive strategies and efforts to improve access to timely, high-quality stroke care. Addressing these inequities requires targeted public health initiatives, enhanced stroke prevention programs, and improved access to acute stroke care in high-risk communities.

The COVID-19 pandemic disrupted healthcare systems, adding complexities to stroke care [12,13]. COVID-19 has been associated with both acute and chronic neurological manifestations [14,15,16], and is recognized as an independent risk factor for AIS, further complicating management and outcomes during the pandemic [17]. Specifically, the pandemic has significantly disrupted stroke treatment pathways, leading to prolonged hospitalizations, decreased utilization of reperfusion therapy, and delays in-hospital workflow [13].

Given annual variations in AIS hospitalization, demographic trends, treatment patterns, and outcomes in the U.S., effective strategies are crucial, especially during public health crises. Understanding how the COVID-19 pandemic has affected stroke incidence, treatment access, and patient outcomes is vital for guiding future preparedness efforts. This study utilizes National Inpatient Sample (NIS) data from 2016 to 2021, covering both pre-pandemic and peri-COVID-19 periods. It examines the shifts in stroke care patterns induced by the pandemic, changes in demographic incidence, and the impacts on outcomes, aiming to provide insights for future interventions to tackle stroke care challenges in the U.S.

## 2. Materials and Methods

### 2.1. Data Source

The National Inpatient Sample (NIS) is the largest all-payer longitudinal inpatient database in the U.S., maintained by the Healthcare Cost and Utilization Project (HCUP) under the Agency for Healthcare Research and Quality (AHRQ) [18].

NIS includes a 20% stratified sample of hospital discharges nationwide, categorized by hospital characteristics (size, region, urban/rural location, teaching status, and ownership) [19,20]. Because the data are publicly available and de-identified, institutional review board approval and patient consent were not required. The NIS transitioned from ICD-9 to ICD-10 coding in 2016, and the latest data available for this study was from 2021 [20,21].

### 2.2. Patient Selection and Inclusion/Exclusion Criteria

**Figure 1:**
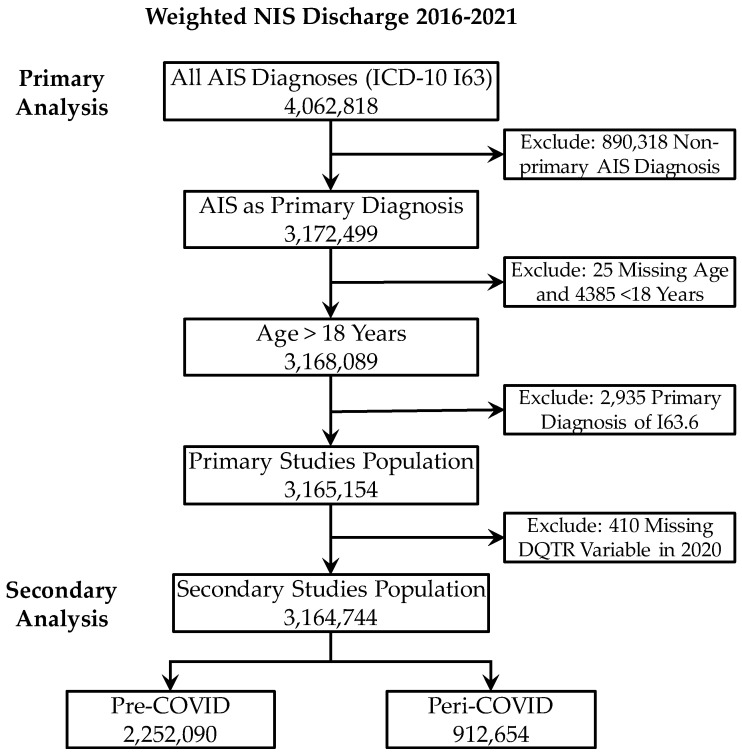
Study data selection flowchart.

Adults (age ≥18) with a primary diagnosis of acute ischemic stroke (AIS) of arterial origin were identified using ICD-10-CM codes I63. Exclusion criteria were patients with ICD-10-CM code I63.6 (for cerebral venous sinus thrombosis). The data selection flowchart is shown in Figure 1.

### 2.3. Demographics and Clinical Data

We analyzed yearly trends for demographic data (age, gender, race), hospital type (e.g., teaching vs. non-teaching), and clinical variables (risk factors, treatments like intravenous thrombolysis (IVT), and mechanical thrombectomy (MT) for AIS admissions. A secondary analysis separated data into pre- and peri-COVID-19 groups, with pre-COVID-19 defined as 2016–2020 (Q1) and peri-COVID-19 as 2020 (Q2)–2021, based on the Discharge Quarter (DQTR) variable. Hospitalization costs were estimated by multiplying NIS total charges by annual cost-to-charge ratios (CCR), adjusted for inflation using the 2021 Consumer Price Index [22]. Incidence calculations used July U.S. population estimates for each study year [23]. A detailed list of ICD-10 codes used for the analysis can be found in Supplement Table S1. Stroke risk factors, including COVID-19, were identified via ICD-10 codes (Supplement Table S1) to assess their effect on incidence and outcomes.

### 2.4. Primary Outcome

The primary outcome was trends in discharge disposition for ischemic stroke admissions from 2016 to 2021, analyzed separately for pre-COVID-19 (2016–2019) and peri-COVID-19 (2020–2021) periods. Discharge categories included routine discharge, transfers to short-term hospitals or other facilities (e.g., long-term care, rehabilitation), home health care, discharge against medical advice, and in-hospital death. Discharge definitions are available on the AHRQ website [https://hcup-us.ahrq.gov/db/vars/dispuniform/nisnote.jsp, accessed on 10 January 2025]. 

### 2.5. Secondary Outcomes

Secondary outcomes included trends in intracerebral hemorrhage (ICH), subarachnoid hemorrhage (SAH), intubation, mechanical ventilation rates, in-hospital deaths, and complications (e.g., cerebral edema, brain herniation, paralysis, neglect, aphasia) pre- and peri-COVID. Complications were identified via a stroke severity scale using NIS data [24]. The National Institutes of Health Stroke Scale (NIHSS) categorized stroke severity when available: mild (NIHSS 0–5), moderate (NIHSS 6–15), and severe (NIHSS ≥ 16). In the year 2016, NIHSS was not recorded for NIS database. Relevant ICD-10 codes were used to maintain alignment with validated definitions, though administrative data may limit accuracy for some clinical outcomes. We assessed the potential impact of COVID-19 on stroke outcomes, as disruptions, delayed care, and altered protocols during the pandemic might have influenced treatment and recovery.

### 2.6. Statistical Analysis

Descriptive statistics were used to generate national estimates, with all analyses conducted in SAS 9.4M7 and SAS Studio 3.81 (SAS Institute, Cary, NC, USA), accommodating NIS’s complex survey design. The ‘SURVEYMEANS’, ‘SURVEYFREQ’, and ‘SURVEYREG’ procedures accounted for stratification, clustering, and weighting. Missing data were handled with the NOMCAR (not missing completely at random) option, including all observations in variance estimation, which assumes missingness relates to observed data. Subgroup analyses used the DOMAIN function within survey procedures to examine trends across specific populations. 

Categorical variables were analyzed using the Rao-Scott χ^2^ test, which adjusts for complex survey design, to identify significant associations between categories. Yearly trends in continuous variables (e.g., age, length of stay, and adjusted cost) were assessed using the ‘SURVEYREG’ procedure, applying stratification, clustering, and weighting for robust trend estimation. Statistical significance was set at *p* < 0.05.

## 3. Results

From 2016 to 2021, 3,165,154 weighted hospital discharge records met inclusion criteria. The mean patient age was 70.0 ± 0.03 years, with a mean hospital stay of 5.1 ± 0.01 days, and an average cost of $16,765 ± 71. Men accounted for 50.5% of AIS cases.

### 3.1. Demographics and Temporal Trends

The AIS cases increased from 507,920 in 2016 to 535,694 in 2021. During this time, the male proportion rose from 49.8% to 51.4%, while the female proportion decreased from 50.2% to 48.6% (Table 1). White patients were the majority but declined from 68.5% to 66.8%. Black, Hispanic, and Asian/Pacific Islander cases saw minor increases, while Native American representation remained low (0.3–0.4%). These results were not statistically significant (Table 1). AIS was more frequent among White (68.0%) and Black (17.5%) populations than in the general population.

Over 2016–2021, 4.0% of AIS patients died in-hospital, 40.4% were transferred to another facility, 35.8% had routine discharges, and 15.8% went to home health care. Medicare covered 64.6% of cases, with 30.9% from the lowest income quartile. AIS admissions peaked in 2019 (17.4%) and were most prevalent in the South (42.4%), predominantly at urban teaching hospitals (73.1%) (Table 2). NIHSS data had varying regional missing rates, the highest in the South (42.5%) (Table 1).

The mean AIS patient age declined from 70.3 ± 0.1 in 2016 to 69.7 ± 0.1 in 2020 and 2021 (*p* = 0.0195, *p* < 0.0001). Cases among ages 18–49 and 60–79 rose, while ages 50–59 and over 80 decreased (Table 1). The pre-COVID-19 (2016–2019) to peri-COVID-19 (2020–2021) analysis reflected similar trends, with a decrease in mean age from 70.2 ± 0.04 years pre-COVID-19 to 69.6 ± 0.05 years peri-COVID-19 (*p* < 0.0001). During the peri-COVID-19 period, White admissions declined, while Black, Hispanic, and Asian/Pacific Islander admissions slightly rose, though not significantly (Table 3). Rural and urban non-teaching hospital admissions declined, while urban teaching hospital admissions increased (Table 2).

### 3.2. Reperfusion Therapy Utilization, Intubation, and Complication Rates

**Figure 2:**
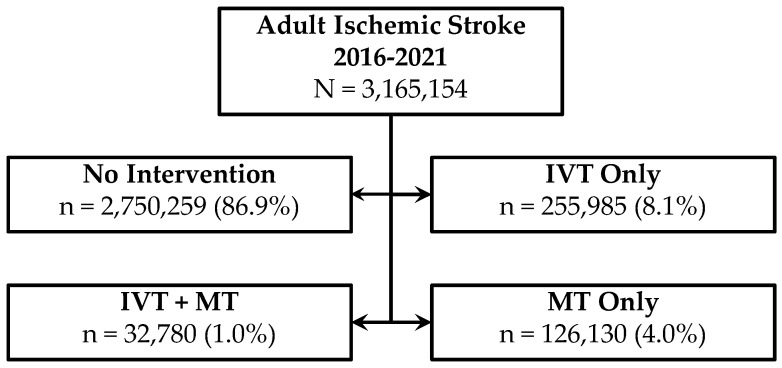
Total number of AIS admissions in 6-year span and intervention/no intervention groups. Abbreviations: IVT, intravenous thrombolysis; MT, mechanical thrombectomy.

From 2016 to 2021, 8.1% of AIS patients received IVT alone, 1% received both IVT and MT, and 4% received MT alone (Figure 2). MT use rose from 2.2% of cases in 2016 to 5.6% in 2021, while IVT and MT combined increased from 0.7% to 1.2%. Intubation and ventilation rates grew from 4.8% pre-COVID-19 to 5.5% peri-COVID-19 (*p* < 0.0001), with 12.4% of intubated patients having COVID-19, compared to 5.4% without COVID-19 (Table 1, Table 2 and Table 3).

ICH as a complication in MT-only cases increased from 16% in 2016, peaking at 19.6% in 2019, and declined to 19.2% in 2021 (*p* = 0.0015). For IVT-only patients, ICH complications peaked at 7.2% in 2018 and fell to 6.7% by 2021 (*p* < 0.0001). SAH and ICH complications were not statistically significant in patients who received combined IVT and MT (Supplement Table S2).

Complications such as neglect (2.2% vs. 3.2%), brain herniation (1.6% vs. 2.2%), cerebral edema (5.0% vs. 6.2%), paralysis (6.7% vs. 7.2%), and aphasia (21.7% vs. 23.4%) increased during the peri-COVID-19 period (*p* < 0.0001) (Table 3).

ICH rates among transferred patients were highest for MT-only cases, decreasing from 46.7% in 2016 to 41.3% in 2021. The combined MT and IVT group peaked at a 10.8% ICH rate in 2017, dropping to 7.8% in 2021. The IVT-only group had its highest ICH rate in 2017 (7.2%), declining to 4.6% by 2021 (Supplement Table S3). The MT-only group had the highest SAH rates among transfers, peaking at 52% in 2017, then falling to 38.2% in 2021. The non-intervention group’s SAH rates increased from 34.3% in 2016 to 37.9% in 2021 (Supplement Table S3).

### 3.3. Risk Factor Trends

AIS admissions showed decreased rates of hypertension, family history of stroke, peripheral vascular disease, and coronary artery disease over six years. Conversely, admissions with diabetes, obesity, atherosclerosis, hyperlipidemia, atrial flutter, hypercoagulable states, and myocardial infarction increased over time (Table 4). Atrial fibrillation peaked in 2018 and declined, with higher rates pre-COVID-19 than peri-COVID-19 (Table 4).

### 3.4. Hospital and Regional Characteristics, Stroke Incidence, and In-Hospital Mortality

AIS admission rates showed no significant regional differences, though prevalence was highest in the South and lowest in the West. In-hospital death rates were highest in the Northeast and lowest in the South. Nationwide, stroke incidence rose until 2019, peaked, declined in 2020, and rose in 2021, remaining below the 2019 peak—a trend observed in all regions (Supplement Figure S1).

In-hospital mortality decreased from 2016 to 2020 but rose in 2021, exceeding previous years. Regionally, the Northeast had declining mortality until 2019, then an increase in 2020, peaking in 2021. The Midwest had fluctuations, with its lowest in 2020, but rose to 2016 levels by 2021. The South’s mortality fell until 2020, with its highest rate in 2021. Mortality in the West increased, peaking in 2021 (Supplement Figure S2).

**Figure 3:**
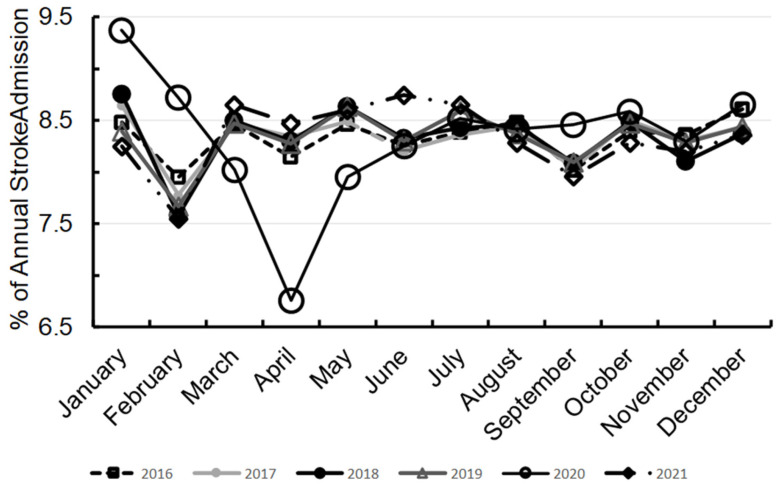
Monthly percent of annual stroke admissions in the US between 2016 and 2021.

In 2020, January had the highest admissions at 9.4% and April had the lowest at 6.8%. By 2021, admissions returned to pre-pandemic levels, with March, June, and July each at 8.7% of admissions (Figure 3).

### 3.5. Discharge Disposition and NIHSS Score

Routine care discharges increased until 2019, then declined, although 2020 and 2021 absolute discharges exceeded 2016 levels. Discharges to short-term hospitals decreased, while transfers to other facilities declined over six years. Home health care discharges and patients leaving against medical advice rose. In-hospital mortality dropped until 2019 but rose to 4.2% in 2021, matching 2016 rates (Table 2). Length of stay and costs increased peri-COVID-19 (Table 3).

Discharge trends between pre- and peri-COVID-19 periods showed a significant decrease in Medicare recipients and an increase in Medicaid and private insurance (Table 3). NIHSS score data, available from 2017, indicated shifts in stroke severity: mild strokes (NIHSS ≤5) increased from 57.7% to 62.4%, while moderate (NIHSS 6–15) and severe strokes (NIHSS ≥16) decreased from 27.7% to 25.0% and 14.5% to 12.6%, respectively (Table 1). The mean NIHSS score fell from 7.1 ± 0.1 to 6.4 ± 0.1. Comparing pre- to peri-COVID-19 periods, NIHSS scores also declined from 6.7 ± 0.03 to 6.4 ± 0.05 (Table 3). NIHSS data had a 49.1% missing rate.

## 4. Discussion

Our study highlights significant changes in AIS admissions during the COVID-19 pandemic. Admissions decreased in 2020 compared to 2019, aligning with studies showing reduced stroke presentations during the pandemic’s early phase [25,26,27]. From 2016 to 2019, stroke incidence rose annually, while the death rate declined, likely due to better treatments and increased public awareness. This decrease in 2020 may reflect delays in seeking care due to lockdowns, fear of infection, and overwhelmed healthcare systems. AIS admissions rose again in 2021, possibly due to either a true rise in incidence or improved healthcare-seeking behaviors despite ongoing restrictions, which aligns with studies showing a recovery in stroke admissions post-pandemic [28,29]. Regional differences in stroke incidence and outcomes, particularly higher rates in the South, may be attributed to socioeconomic factors and healthcare practices [30], consistent with prior findings of regional disparities in stroke outcomes and care access [31,32].

Our analysis reveals notable demographic shifts in AIS patients during the pandemic. AIS admissions among the elderly (aged 80 and above) decreased, likely due to COVID-19 exposure fears and their higher risk for severe outcomes [33,34,35,36]. This aligns with reports indicating that older adults were more likely to delay seeking hospital care. Conversely, AIS admissions rose among younger adults aged 18–49 and middle-aged individuals aged 60–79, potentially driven by rising stroke risk factors like obesity, diabetes, and hypertension in these groups, mirroring national trends [37]. It is possible that for patients aged 18–49, lifestyle changes, COVID-19 infection, or changes in healthcare-seeking behavior might contribute to increased stroke incidence based on an example of a prior study [38]. Despite the decline in stroke admissions among Native Americans, this may reflect their smaller population size and limited healthcare access rather than a true reduction in incidence [39]. We observed a consistent reduction in stroke admission rates from January to February each year, except in 2020, when rates continued to decline until April, which marked the study period’s lowest monthly rate (Figure 2). This drop likely reflects the impacts of lockdowns, infection fear, and overwhelmed healthcare systems [40]. The highest 2020 stroke admission rate occurred in January, potentially due to cold weather and early COVID-19 circulation, which could induce hypercoagulability [41,42]. Delayed care during the December 2019 holidays may also have influenced early 2020 admissions. By May 2020, admission rates normalized as healthcare access improved with easing lockdowns.

Regarding stroke complications, we noted an increasing trend in hemorrhagic complications (ICH and SAH) among all AIS admissions, potentially linked to the increased use of MT and combined MT with IVT therapies, which carry a risk of intracranial bleeding [43,44]. Higher rates of these complications were observed in patients undergoing MT alone without IVT or no intervention each year, possibly due to the deployment of MT in patients with extensive strokes, associated with an increased risk of hemorrhage (Supplement Table S2) [45]. A notable trend was the consistent decline in ICH rates among transferred patients from 2016 to 2021 across all intervention groups. SAH rates among transferred patients also showed a decreasing trend during the same period. Rates of cerebral edema, neglect, brain herniation, and aphasia were higher during the pandemic, likely due to delayed hospital admissions resulting in larger infarcted brain tissue and more severe neurological deficits [46,47].

We observed increased MT use during the pandemic, indicating a shift towards more aggressive stroke management, supported by MT’s efficacy [48,49,50,51]. This increase may be tied to advancements in MT technology, updated clinical guidelines, and evidence supporting MT’s superiority over conventional treatments [48,49,50]. COVID-19-associated hypercoagulability may have further influenced this shift [42,52], aligning with national data showing an overall rise in endovascular therapies in recent years [5]. However, a significant proportion of AIS patients did not receive any intervention, likely due to delayed care-seeking or milder stroke severity, highlighting ongoing challenges in timely stroke management.

Our study shows a significant increase in mechanical ventilation rates among stroke patients during the pandemic, likely due to respiratory complications from COVID-19 and the use of general anesthesia (GA) for MT procedures [53,54,55,56,57]. The rise in intubation and ventilation rates since 2019 suggests changes in clinical practices or increased procedural complexity [58,59]. Increased complications, such as hemorrhagic events and cerebral edema, may be linked to higher MT usage and delays in care, resulting in larger infarct sizes and more severe outcomes [43,44,46,47]. This is supported by studies noting a rise in procedure-related complications during the pandemic [60]. Notably, the increase in intubation rates began in 2019, indicating other contributing factors beyond the pandemic, though the exact reasons remain unclear. 

Risk factor trends reveal both improvements and concerns. The decrease in traditional risk factors like hypertension and coronary artery disease reflects better management of them [61,62]. However, increases in diabetes and obesity during the pandemic are troubling and may be linked to lifestyle changes [63,64,65,66,67]. Alcohol consumption rose in the pandemic’s first year, correlating with increased atrial flutter risk [67,68]. COVID-19 has been shown to exacerbate stroke risk through hypercoagulability and inflammation [17,42,69]. Additionally, COVID-19-associated coagulopathy, with lower protein C levels and antiphospholipid antibodies, heightens stroke risk [70]. Our study found an increase in recorded hypercoagulability cases from the peri- to pre-COVID-19 periods.

Fluctuations in stroke incidence and in-hospital mortality from 2016 to 2021 seem significantly influenced by the pandemic. The rise in incidence from 2016 to 2019 is attributed to factors like an aging population, increasing stroke risk factors, and improved detection [3]. The 2020 drop in incidence may reflect healthcare disruptions, such as overwhelmed emergency departments and restricted access, contributing to underreporting of strokes [71]. The 2021 incidence increase may result from vaccination campaigns, public health messaging encouraging emergent care for new symptoms [72], and expanded telemedicine use alongside modified hospital protocols and enhanced telemedicine use and modified hospital protocols [72,73].

The in-hospital mortality decline from 2016 to 2020 suggests advances in stroke care, including better acute and post-stroke management [74]. The 2021 mortality increase likely resulted from delayed patient presentations, leading to worse outcomes and higher mortality [75]. Additionally, more patients may have presented after the 2020 drop, as our analysis suggests.

Regional variations in stroke incidence and hospital characteristics were also observed, with the South showing higher AIS rates, possibly due to inflammation, lifestyle choices, and socioeconomic disparities [30]. The increase in hemorrhagic complications and MT rates may reflect regional healthcare resource levels and patient management practices. Differences in-hospital types, such as rural versus urban and teaching versus non-teaching hospitals, influenced stroke admissions. Rural and non-teaching hospitals saw declines, possibly due to accessibility changes, while urban and teaching hospitals saw increases due to better resources [76,77].

As shown in Supplement Table S3, high ICH, and SAH rates were consistently observed in patients transferred for MT, reflecting longer reperfusion times [78]. SAH and ICH rates among MT patients show a decline over the six-year period, reflecting improvements likely due to increased MT experience and improved procedural systems [79].

The increased proportion of patients discharged to home or home healthcare during the pandemic suggests a shift toward outpatient management, potentially due to reduced hospital capacity or patient preference [57]. NIHSS scores indicate an increase in stroke severity during this period, though missing data, particularly in the South, limits severity assessment and outcome analysis [80,81]. This missing data may result from regional recording practices and healthcare constraints [80,81]. The increased severity of strokes could also be linked to delayed care-seeking, allowing strokes to progress [39,82,83,84]. These trends underscore the need for improved data collection to better understand stroke outcomes. Inconsistent NIHSS documentation in large databases impedes severity trend evaluations and their implications. It is possible that different types of strokes were underreported in-hospital records (given the high number of missed NIHSS measurements), leading to an apparent decline in their incidence. As a result, stroke severity may seem lower during COVID-19, while in reality, more severe cases were not properly documented. Improved data collection and reporting will enhance our understanding of stroke outcomes and inform targeted interventions.

Rising healthcare costs in the U.S. have become a major concern, particularly highlighted by our analysis of increasing hospitalization costs for AIS patients during the COVID-19 pandemic [85,86]. Contributing factors include longer hospital stays, the necessity for protective measures, and greater use of critical care resources. Patients receiving MT or IVT faced significantly higher costs than those who did not [87].

Adjusted for inflation, average hospitalization costs during the peri-COVID-19 period were markedly higher than in the pre-COVID-19 period, reflecting the complexities of treatment protocols and the strain on healthcare systems during the pandemic. Hospitals encountered elevated operational costs due to the demand for personal protective equipment, specialized staffing, and intensive care services.

These rising costs emphasize the pandemic’s broader economic burden on healthcare systems and raise concerns about the long-term sustainability of stroke care, especially for patients needing advanced interventions like MT and IVT. Future research should focus on strategies to optimize resource management and mitigate the financial impact of acute stroke care during health crises.

## 5. Study Limitations

There are several limitations to consider in interpreting our findings. One limitation is our reliance on accurate ICD-10 codes for identifying patients. While ICD-10 generally has a high predictive value for AIS, inaccuracies can occur [88].

For instance, one study showed that ICD-10 codes correctly identified 98% of IVT patients and 87% of MT patients [89], but errors in complex cases remain possible. Another limitation is that NIS hospital discharge data do not specify clinical indications for MT, hindering our ability to determine whether the rise in MT cases reflects the inclusion of patients presenting later, as newer guidelines permit. This lack of detail limits our assessment of changing stroke management protocols’ impact on treatment trends and outcomes.

Additionally, NIS does not indicate whether hospitals are comprehensive stroke centers (CSCs) or thrombectomy-capable stroke centers (TSCs). Anonymized hospital identifiers prevent hospital-level analyses, limiting the evaluation of stroke center designations’ impact on therapy use and outcomes. The lack of standardized discharge disposition data also complicates outcome interpretation, as terms like “home health care” can vary by state.

The NIS database’s lack of time-sensitive data, such as the interval from symptom onset to hospital presentation, prevents analysis of patients presenting within critical reperfusion therapy time windows. NIS-based studies are also retrospective, relying on previously collected data, and limiting causal inferences. The NIS excludes outpatient or emergency department-treated patients, impacting the generalizability of findings to include milder strokes or TIAs. 

Finally, the absence of thrombolysis in the cerebral infarction scale, imaging results, and modified Rankin scale at follow-up further limits our study. While NIHSS measurements are reported in the NIS, the reporting rates are poor and significantly differ across age groups. These clinical metrics are essential for assessing stroke severity, treatment efficacy, and long-term outcomes.

## 6. Conclusions

This analysis of AIS trends from 2016 to 2021 reveals significant changes during the COVID-19 pandemic, with shifts in admissions, demographics, treatment patterns, and outcomes. 

The findings from this study underscore the importance of timely stroke care and intervention, especially during public health crises. Healthcare providers must remain vigilant in recognizing stroke symptoms and ensuring patients receive timely care. Hospitals and stroke centers should prioritize the establishment of protocols to manage patient flow and ensure access to acute stroke therapies during emergencies.

Furthermore, continuing education and training for healthcare professionals in stroke recognition and management, particularly in the context of evolving guidelines and technologies, are essential. Engaging with community partners to promote awareness of stroke symptoms and the importance of seeking immediate care can help mitigate the impact of future crises on stroke care delivery.

Overall, while our analysis highlights the challenges presented by the COVID-19 pandemic, it also points to opportunities for improving stroke care systems. Emphasizing evidence-based practices, advancing stroke research, and prioritizing health equity will be critical in navigating the ongoing landscape of stroke care in a post-pandemic world.

## Figures and Tables

**Table 1:** Patient demographics and reperfusion therapy choice, NIHSS values from 2016 to 2021 period.

Variables	2016 (n = 507,920)	2017 (n = 523,340)	2018 (n = 532,050)	2019 (n = 551,895)	2020 (n = 514,255)	2021 (n = 535,694)	*p* Value
**Age**							<0.0001
18–49	7.9%	7.9%	7.9%	8.0%	8.2%	8.2%	
50–59	14.9%	14.9%	14.7%	14.5%	14.7%	14.3%	
60–69	22.7%	22.8%	23.0%	23.4%	23.9%	24.2%	
70–79	23.8%	24.3%	24.9%	25.1%	25.6%	26.1%	
80+	30.7%	30.1%	29.5%	29.0%	27.6%	27.2%	
**Sex**							<0.0001
Woman	50.6%	50.1%	49.5%	49.2%	49.1%	48.6%	
Man	49.4%	49.9%	50.5%	50.8%	50.9%	51.4%	
**Race**							0.5734
White	69.5%	68.5%	67.9%	68.0%	67.4%	66.9%	
Black	16.9%	17.4%	17.2%	17.6%	18.0%	17.8%	
Hispanic	7.7%	8.2%	8.9%	8.2%	8.3%	8.8%	
Asian/PI	2.9%	3.0%	3.1%	3.1%	3.1%	3.3%	
Native American	0.5%	0.4%	0.5%	0.5%	0.5%	0.5%	
Other	2.6%	2.6%	2.5%	2.6%	2.8%	2.7%	
**Procedure choice**							<0.0001
Neither	89.8%	88.6%	86.7%	86.0%	85.3%	85.0%	
MT only	2.2%	2.6%	3.8%	4.4%	5.2%	5.6%	
MT + IVT	0.7%	0.8%	1.0%	1.2%	1.3%	1.2%	
IVT only	7.3%	7.9%	8.4%	8.4%	8.2%	8.2%	
**NIHSS**							<0.0001
Mild: ≤5	N/A	57.7%	60.3%	62.3%	62.1%	62.4%	
Moderate: 6–15	N/A	27.7%	26.1%	25.1%	25.2%	25.0%	
Severe: 16+	N/A	14.5%	13.7%	12.6%	12.7%	12.6%	
Mean ± SEM	N/A	7.1 ± 0.1	6.8 ± 0.1 *	6.4 ± 0.1 *	6.5 ± 0.1 *	6.4 ± 0.1 *	<0.0001

Abbreviations: PI, pacific islander; MT, mechanical thrombectomy; IVT, intravenous thrombolysis; NIHSS, National Institutes of Health Stroke Scale. * Statistically significant when compared to 2017.

**Table 2:** Hospital region/status, discharge status, intubation status, and complications from 2016 to 2021 period.

Variables	2016 (n = 507,920)	2017 (n = 523,340)	2018 (n = 532,050)	2019 (n = 551,895)	2020 (n = 514,255)	2021 (n = 535,694)	*p* Value
**Hospital Region**							1.0
Northeast	17.8%	17.5%	17.3%	17.2%	16.9%	17.1%	
Midwest	21.8%	21.7%	21.7%	21.5%	21.5%	21.4%	
South	41.8%	42.2%	42.3%	42.6%	42.6%	42.7%	
West	18.5%	18.6%	18.6%	18.7%	19.0%	18.9%	
**Hospital status**							<0.0001
Rural	8.1%	7.8%	7.2%	7.0%	7.2%	7.2%	
Urban non-teaching	25.5%	22.1%	19.3%	16.9%	16.9%	16.6%	
Urban teaching	66.4%	70.0%	73.5%	76.1%	75.9%	76.2%	
**Discharge status**							<0.0001
Routine	35.6%	35.7%	35.9%	36.3%	35.6%	35.9%	
Short-term hospital	2.9%	2.9%	2.8%	2.8%	2.6%	2.4%	
Other type of facility	42.5%	41.8%	41.7%	41.1%	37.7%	37.8%	
Home health care	13.8%	14.6%	14.7%	15.0%	18.8%	18.2%	
Against medical advice	1.0%	1.0%	1.0%	1.0%	1.3%	1.5%	
Died in-hospital	4.2%	4.0%	3.9%	3.7%	4.0%	4.2%	
LOS, days (mean ± SEM)	4.9 ± 0.04	4.9 + 0.04	4.9 ± 0.04	5.0 ± 0.04 **	5.2 ± 0.04 **	5.5 ± 0.05 **	<0.0001
Cost ^ψ^ (mean ± SEM)	$15,167 ± 151	$15,599 ± 156	$15,975 ± 154 **	$16,989 ± 178 **	$18,317 ± 197 **	$18,482 ± 193 **	<0.0001
**Complications**							
Intubation/Mechanical ventilation	4.6%	4.6%	4.9%	5.1%	5.6%	5.5%	<0.0001
SAH	0.6%	0.6%	0.8%	0.9%	1%	1.1%	<0.0001
ICH	2.9%	3.3%	4.5%	4.7%	5.1%	5.3%	<0.0001

Abbreviations: SAH, subarachnoid hemorrhage; ICH, intracranial hemorrhage ** Statistically significant when compared to 2016. **^ψ^** Adjusted to 2021 Consumer Price Index.

**Table 3:** Patient demographics, payer status, discharge status, and intubation/mechanical ventilation for pre- and peri-COVID-19 periods.

Variables	Pre-COVID-19 (2016–2019)	Peri-COVID-19 (2020–2021)	*p* Value
Number of admissions	2,252,090	912,654	
**Sex**			<0.0001
Woman	49.8%	48.8%	
Man	50.2%	51.2%	
**Age Group**			<0.0001
18–49	7.9%	8.3%	
50–59	14.7%	14.5%	
60–69	23.0%	24.1%	
70–79	24.6%	25.9%	
80+	29.8%	27.2%	
**Race/Ethnicity**			0.1032
White	68.4%	67.1%	
Black	17.3%	17.9%	
Hispanic	8.3%	8.6%	
Asian/Pacific Islander	3.0%	3.2%	
Native American	0.5%	0.5%	
Other	2.6%	2.7%	
**Procedure choice**			<0.0001
Neither MT nor IVT	87.6%	85.2%	
MT only	3.4%	5.5%	
MT + IVT	1%	1.2%	
IVT only	8.1%	8.1%	
**Payer**			<0.0001
Medicare	65.3%	63%	
Medicaid	9.2%	10.4%	
Private Insurance	18.9%	19.4%	
Other	6.6%	7.3%	
**Discharge Status**			<0.0001
Routine	35.8%	35.9%	
Short-term hospital	2.8%	2.5%	
Other type of facility	41.7%	37.2%	
Home health care	14.6%	18.9%	
Against Medical Advice	1.0%	1.4%	
Died in-hospital	4.0%	4.1%	
LOS (mean ± SEM)	5.0 ± 0.02	5.3 ± 0.04	<0.0001
Cost (mean ± SEM) *	$16,094 ± 76.30	$18,419 ± 139.30	<0.0001
**Complications**			
Intubation/Mechanical ventilation rate	4.8%	5.6%	<0.0001
Neglect	2.2%	3.2%	<0.0001
Cerebral Edema	5%	6.2%	<0.0001
Brain Herniation	1.6%	2.2%	<0.0001
Aphasia	6.7%	7.2%	<0.0001
NIHSS			<0.0001
Mild: ≤5	60.7	62.4	
Moderate: 6–15	26.0	24.9	
Severe: 16+	13.3	12.7	
**Mean NIHSS ± SEM**	6.7 ± 0.03	6.4 ± 0.05	<0.0001

Abbreviations: LOS, length of stay; MT, mechanical thrombectomy; IVT, intervenors thrombolysis; NIHSS, National Institute of Health stroke scale. * Adjusted to 2021 Consumer Price Index.

**Table 4:** Risk factor distribution pre- and peri-COVID-19 and yearly.

Risk Factors	Pre-COVID (2016–2019)	Peri-COVID (2020–2021)	*p* Value	2016	2017	2018	2019	2020	2021	*p* Value
Admission number	2,252,090	912,654	NA	507,920	523,340	532,050	551,895	514,255	535,694	NA
Hypertension	58.8%	55.7%	<0.0001	66.1%	57.7%	56.8%	55.9%	55.7%	55.6%	<0.0001
Tobacco use	20.1%	20.4%	0.1049	20.0%	20.1%	20.2%	20.3%	20.4%	20.3%	0.7174
Diabetes	38.9%	40.1%	<0.0001	38.1%	38.7%	39.3%	39.3%	39.9%	40.2%	<0.0001
Family history of Stroke	3.6%	3.2%	<0.0001	4.0%	3.7%	3.5%	3.4%	3.2%	3.1%	<0.0001
TIA History	15.4%	15.6%	0.2596	15.7%	15.3%	15.2%	15.2%	15.6%	15.6%	0.2262
PVD	4.3%	3.3%	<0.0001	5.7%	4.5%	3.7%	3.6%	3.4%	3.2%	<0.0001
Atherosclerosis	2.5%	2.9%	<0.0001	2.3%	2.4%	2.5%	2.6%	2.8%	2.9%	0.0003
Obesity	13.9%	16.9%	<0.0001	12.5%	13.4%	14.5%	14.9%	16.2%	17.0%	<0.0001
Hyperlipidemia	60.2%	62.7%	<0.0001	59.7%	59.5%	60.0%	60.9%	62.4%	62.8%	<0.0001
Atrial fibrillation	25.1%	24.2%	<0.0001	24.9%	25.2%	25.3%	25.0%	24.2%	24.4%	0.0001
Atrial flutter	2%	2.3%	<0.0001	1.9%	1.9%	2.0%	2.0%	2.3%	2.3%	<0.0001
CAD	23.9%	22.6%	<0.0001	24.4%	24.1%	23.6%	23.6%	23.2%	22.4%	<0.0001
Hypercoagulable state	0.7%	1.1%	<0.0001	0.6%	0.6%	0.7%	0.8%	1.0%	1.3%	<0.0001
MI	2.8%	3.8%	<0.0001	2.4%	2.7%	2.9%	3.1%	3.6%	3.9%	<0.0001

Abbreviations: TIA, Transit ischemic attack; PVD, peripheral vascular disease; MI, myocardial infarction; CAD, coronary artery disease.

## Data Availability

The National Inpatient Sample (NIS) datasets are publicly available. The datasets can be purchased from the AHRQ website (https://hcup-us.ahrq.gov/news/exhibit_booth/nis_brochure.jsp, accessed on 10 January 2025) upon obtaining the HCUP Data Use Agreement (DUA).

## Supplementary Materials

The following supporting information can be downloaded at: https://www.mdpi.com/article/10.3390/jcm14041354/s1, Figure S1: Stroke incidence per 100 K by region. (A) Northeast, (B) Midwest, (C) South, (D) West, and (E) Overall; Figure S2: In-hospital mortality per 100 K by region. (A) Northeast, (B) Midwest, (C) South, (D) West, and (E) Overall; Table S1: ICD-10 codes used for the study.; Table S2: Complication trends by year and procedure group; Table S3: Percent of patients transferred (from both acute care hospitals and other types of health facilities) by year and procedure group.
