# Supplementary material for: Trends in Ischemic Stroke Hospitalization and Outcomes in the United States Pre- and Peri-COVID-19 Pandemic: A National Inpatient Sample Study
Source: J Clin Med. 2025 Feb 18;14(4):1354. doi: 10.3390/jcm14041354 (PMC11856848; doi:10.3390/jcm14041354)
Supplement: Supplementary file 1 [file jcm-14-01354-s001.zip › jcm-3455366-supplementary.pdf]

Journal of Clinical Medicine

MDPI

Supplementary Material

Table S1. ICD-10 codes used for the study.

Variable	ICD-10-CM Codes
Acute Ischemic Stroke (AIS)	I63* except I63.6
**Risk Factors**	
Atrial fibrillation	I48.0, I48.1, I48.2, I48.91
Atrial flutter	I48.3, I48.4, I489.2
COVID	U071, B342, B948, U099, U00*, U49*, U50*, U85*
Hypertension	I1A0, I150, I158, I159, I152, I151, I10*
Diabetes (types 1&2)	E10, E11
Family history of stroke	Z823
History of transient ischemic attack (TIA)	Z8673
TIA	G45
Peripheral vascular disease (PVD)	I739
Intracerebral hemorrhage (ICH)	I61*
Subarachnoid hemorrhage (SAH)	I60*
Atherosclerosis	I70
Obesity/Overweight	E66
Hyperlipidemia	E78
Tobacco use	Z720, F17
Coronary artery disease (CAD)	I251
Other primary thrombophilia	D6859
Myocardial infarction (MI)	I21*, I22*
**Procedures**	
Intravenous thrombolysis (IVT)	3E03317
Mechanical Thrombectomy (MT)	03CG3Z7, 03CH3Z7, 03CJ3Z7, 03CK3Z7, 03CL3Z7, 03CM3Z7, 03CN3Z7, 03CP3Z7, 03CQ3Z7, 03CH3ZZ ,03CJ3ZZ, 03CK3ZZ, 03CL3ZZ, 03CM3ZZ, 03CN3ZZ, 03CP3ZZ, 03CQ3ZZ, 03CG3ZZ
Intubation/Mechanical Ventilation	0BH17EZ, 0BH18EZ, 5A1935Z, 5A1945Z, 5A1955Z
Brain Herniation	G935
Aphasia	R4701
Cerebral edema	G936
Neglect	R414

*All subcodes included.

Table S2. Complication trends by year and procedure group.

Complication type	2016 N (%)	2017 N (%)	2018 N (%)	2019 N (%)	2020 N (%)	2021 N (%)	P value
**Intubation/Mechanical ventilation**							
Neither	17515 (3.8)	17555 (3.8)	17435 (3.8)	17910 (3.8)	17920 (3.9)	17640 (3.9)	0.7966
MT Only	2770 (25.0)	3250 (23.4)	4930 (24.5)	6175 (25.3)	7490 (27.9)	7970 (26.8)	0.0070

MT + IVT	780 (23.4)	1150 (25.0)	1290 (23.6)	1560 (24.1)	1765 (27.0)	1685 (25.8)	0.4270
IVT Only	2065 (5.5)	2230 (5.4)	2435 (5.4)	2380 (5.1)	2175 (5.1)	2270 (5.2)	0.8237
**ICH**							
Neither	10090 (2.2)	12080 (2.6)	15690 (3.4)	16680 (3.5)	16920 (3.9)	18605 (4.1)	<.0001
MT Only	1780 (16.0)	2285 (16.5)	3930 (19.5)	4790 (19.6)	5175 (19.3)	5175 (19.2)	0.0015
MT + IVT	500 (15.0)	700 (15.8)	1050 (19.2)	1065 (16.5)	1090 (16.7)	1170 (17.9)	0.2264
IVT Only	2220 (6.0)	2305 (5.6)	3230 (7.2)	3230 (7.0)	2985 (7.1)	2925 (6.7)	<.0001
**SAH**							
Neither	1650 (0.4)	1765 (0.4)	1840 (0.4)	2140 (0.5)	2365 (0.5)	2715 (0.6)	<.0001
MT Only	625 (5.6)	740 (5.3)	1285 (6.4)	1670 (6.8)	1830 (6.8)	2200 (7.4)	0.0109
MT + IVT	215 (6.4)	275 (6.2)	340 (6.2)	420 (6.5)	485 (7.4)	555 (8.5)	0.1943
IVT Only	400 (1.1)	520 (1.3)	640 (1.4)	735 (1.6)	670 (1.6)	685 (1.6)	0.0315

Table S3. Percent of patient transferred (from both acute care hospitals and other type of health facilities) by year and procedure group.

Complication Type	2016	2017	2018	2019	2020	2021
**Intubation/Mechanical Ventilation**						
Neither	27.3%	30.3%	31.6%	33.1%	33.2%	31.2%
MT Only	36.8%	43.4%	42.2%	38.3%	38.8%	39.1%
MT + IVT	7.1%	6.6%	3.5%	7.1%	4.3%	7.5%
IVT Only	5.1%	6.3%	7.0%	4.6%	6.5%	6.4%
**ICH**						
Neither	31.1%	30.3%	32.6%	31.1%	30.6%	29.4%
MT Only	46.7%	49.4%	44.1%	43.6%	43.4%	41.3%
MT + IVT	10.1%	10.8%	6.3%	8.0%	9.3%	7.8%
IVT Only	3.9%	7.2%	5.0%	4.5%	7.1%	4.6%
**SAH**						
Neither	34.3%	38.0%	32.1%	38.0%	37.3%	37.9%
MT Only	40.2%	52.0%	45.7%	39.6%	41.0%	38.2%
MT + IVT	7.0%	9.1%	7.4%	9.9%	3.6%	1.8%
IVT Only	6.3%	3.8%	6.3%	6.1%	5.2%	5.9%

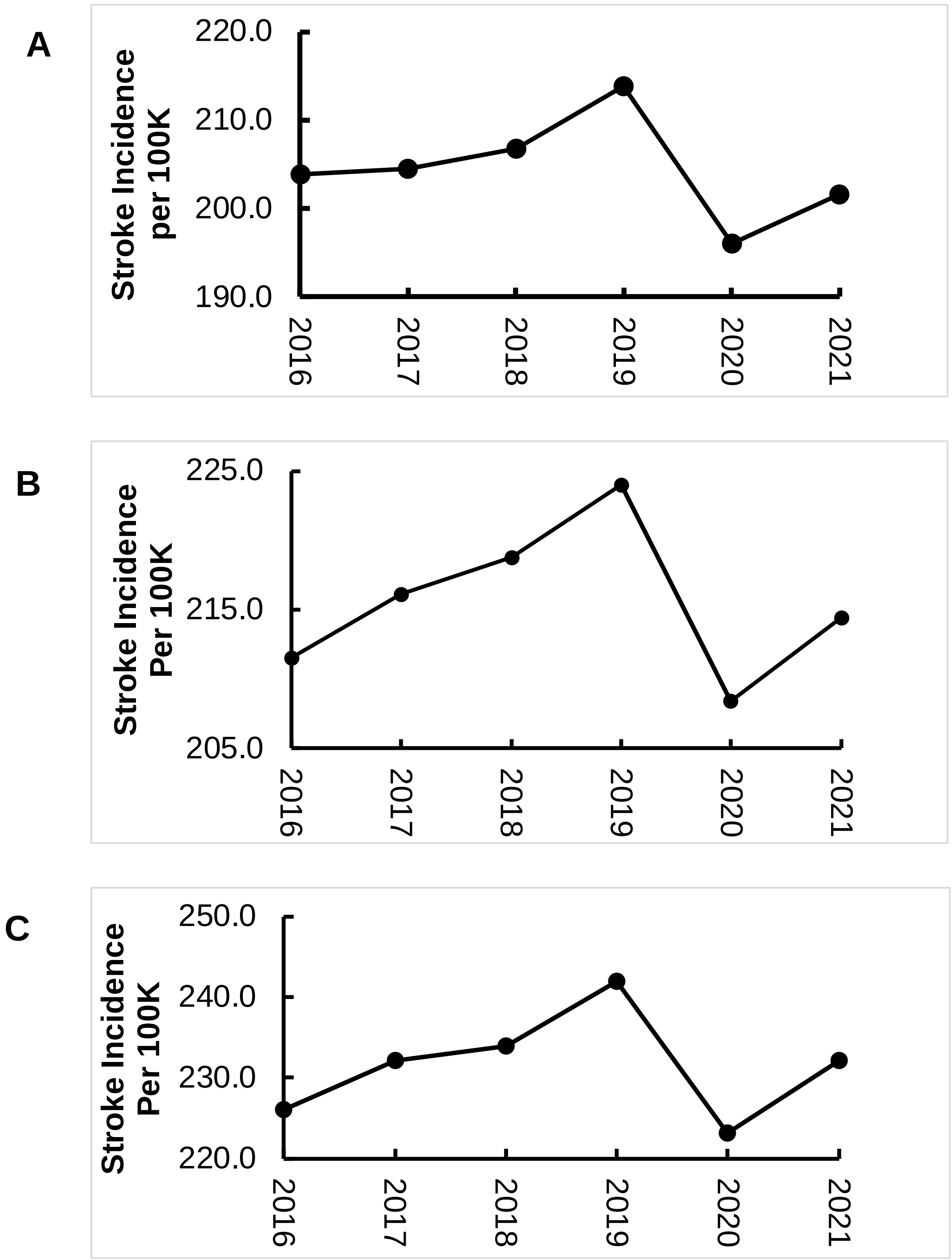
A
Stroke Incidence per 100K
220.0
210.0
200.0
190.0
2016
2017
2018
2019
2020
2021
B
Stroke Incidence Per 100K
225.0
215.0
205.0
2016
2017
2018
2019
2020
2021
C
Stroke Incidence Per 100K
250.0
240.0
230.0
220.0
2016
2017
2018
2019
2020
2021

D

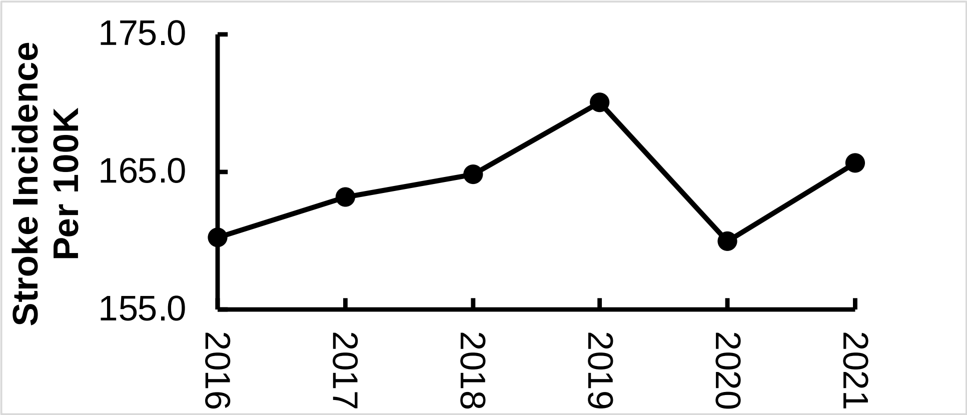

E

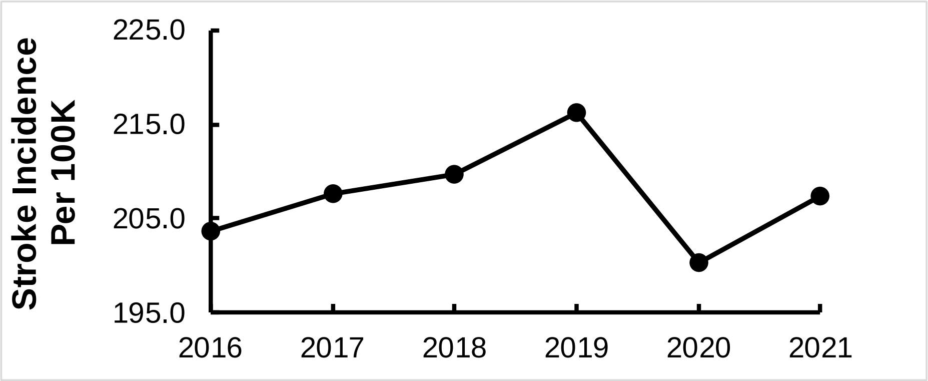

Figure S1. Stroke incidence per 100K by region. A) Northeast, B) Midwest, C) South, D) West, and E) Overall.

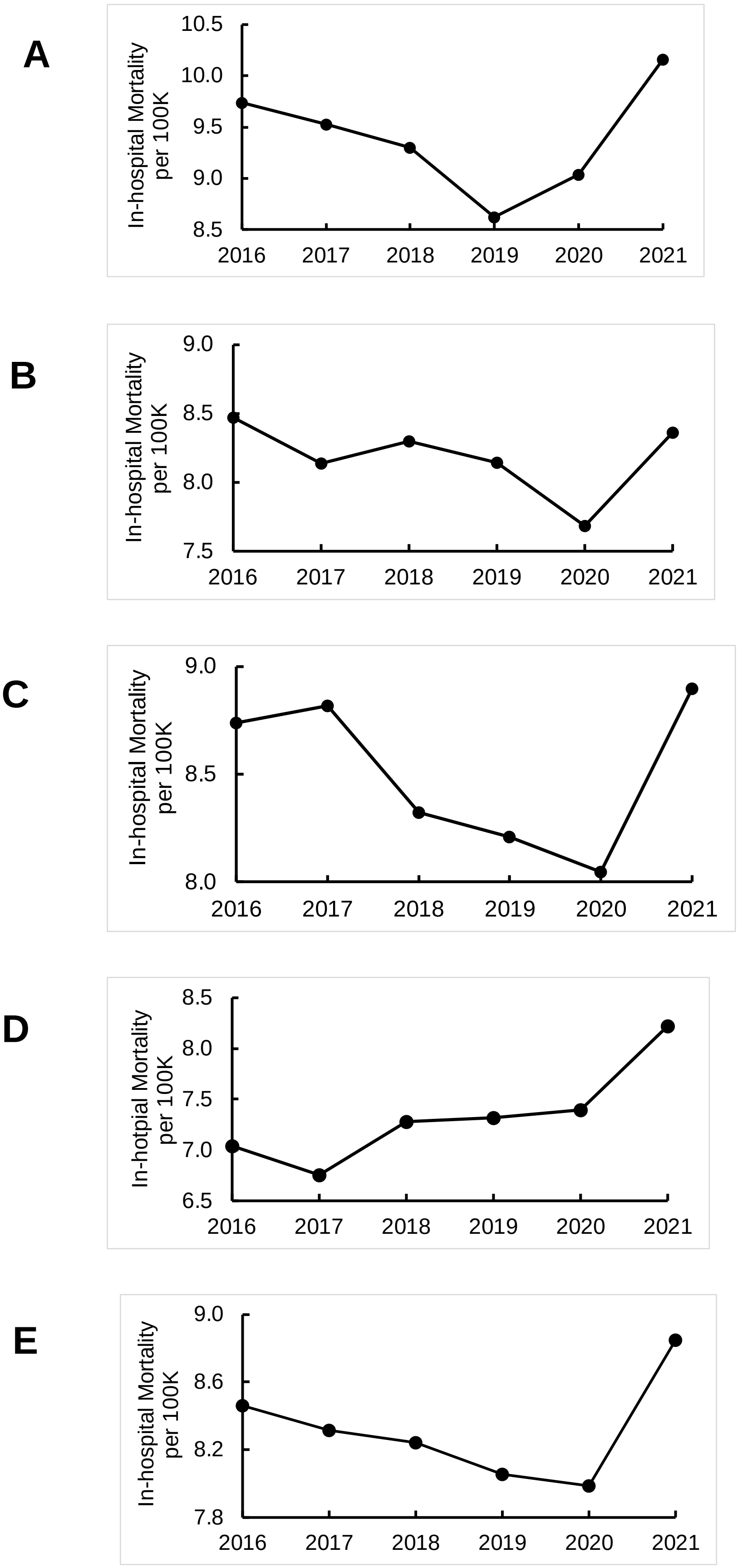

Figure S2. In-hospital mortality per 100K by region. A) Northeast, B) Midwest, C) South, D) West, and E) Overall.
